# Vagus nerve stimulation (VNS) as a long-term adjunctive treatment option in patients with difficult-to-treat depression (DTD)

**DOI:** 10.1192/j.eurpsy.2024.1463

**Published:** 2024-08-27

**Authors:** E. Kavakbasi, H. Bauermeister, L. Lemcke, B. T. Baune

**Affiliations:** ^1^Department of Psychiatry; ^2^Department of Neurosurgery, University Hospital Münster, University of Münster, Münster, Germany; ^3^Department of Psychiatry, Melbourne Medical School, The University of Melbourne, Melbourne; ^4^The Florey Institute of Neuroscience and Mental Health, The University of Melbourne, Parkville, Australia

## Abstract

**Introduction:**

VNS is a long-term adjunctive treatment option in patients with DTD. It has been shown that patients with VNS as add-on to treatment-as-usual (TAU) have higher response and remission rates than TAU alone. Data on the impact of VNS on the other complex concomitant treatments are limited.

**Objectives:**

In this study we evaluated changes in drug load from baseline to 12 months as well as the impact of previous ECT response status at baseline on changes in mean depression severity after 12 months of VNS.

**Methods:**

We included n=20 DTD patients (mean age 52.6 years) in the prospective, observational, naturalistic Restore-Life study, who have been treated with adjunctive VNS as add-on to treatment as usual. The RESTORE-Life study is a multi-center study. In this analysis, we report on exploratory results from a single tertiary center. An index has been calculated for each drug by comparing the actual dose with the standard dose of the drug. The drug load for each patient has been constructed by summing up the indices of all agents prescribed for the patient.

**Results:**

We observed a slight decrease in mean drug load from 4.5 at baseline to 4.4 at 12 months (p=0.594). The drug load was lower in previous ECT-responders than in ECT-non-responders at both timepoints. There was a significant decrease in mean MADRS score from 27.3 at baseline to 15.3 at 12 months (p=0.001). Patients with a history of ECT response at baseline have experienced significantly greater improvement in mean MADRS score at 12 months (p=0.013). Number of maintenance electroconvulsive therapy (ECT) and esketamine sessions decreased from 37 ECT and 58 esketamine sessions in the first six months to 17 ECT (-54%) and 29 esketamine (-50%) sessions between months 6 and 12. VNS-related adverse events were present in 50 % of patients at 12 months (voice alteration/hoarseness 45%, dyspnea and pain during stimulation each 5%). There was no discontinuation of VNS due to adverse events.

**Conclusions:**

Overall, VNS was associated with significant decrease in mean MADRS score at 12 months, whereas we did not detect any significant change in medication load. A more extended observation period might be necessary to observe changes in medication load. There was a reduction in the need of maintenance treatment sessions of ECT and esketamine. History of ECT response may be predictive for greater improvement of depression severity in VNS patients.

**Disclosure of Interest:**

E. Kavakbasi Grant / Research support from: The Sponsor of the Restore-Life study is LivaNova. Our institution received fees from LivaNova for study visits of the Restore-Life study. LivaNova had no influence on the content of this work., H. Bauermeister: None Declared, L. Lemcke: None Declared, B. Baune: None Declared

